# Photoacoustic microscopy reveals deep angiogenic responses in 3D bioprinted tumor–vessel models

**DOI:** 10.1038/s41378-026-01243-y

**Published:** 2026-04-13

**Authors:** Yongjae Jo, Seokgyu Han, Hyunjun Kye, Jinhee Yoo, Junhyung Kim, Inki Kim, Sungsu Park, Byullee Park

**Affiliations:** 1 Department of Biophysics, Institute of Quantum Biophysics, Sungkyunkwan University, Suwon, Republic of Korea; 2 School of Mechanical Engineering, Sungkyunkwan University, Suwon, Republic of Korea; 3 Division of Engineering in Medicine, Department of Medicine, Brigham and Women’s Hospital, Harvard Medical School, Cambridge, MA USA; 4 Departments of MetaBioHealth, Biopharmaceutical Convergence, Sungkyunkwan University, Suwon, Republic of Korea

**Keywords:** Micro-optics, Electrical and electronic engineering

## Abstract

Three-dimensional (3D) tumor–vessel models provide a physiologically relevant platform to study tumor-induced angiogenesis and evaluate therapeutic responses. However, imaging-based analysis of these models is often constrained by limited penetration depth and volumetric resolution. To overcome these challenges, we employed high-resolution photoacoustic microscopy (HR − PAM) to monitor and quantify angiogenesis within bioprinted tumor–vessel models under drug treatments. Compared to confocal microscopy, the PAM achieved a 1.6-fold increase in 1/e² penetration depth, enabling visualization of vascular structures up to ~1 mm in depth. Using our HR − PAM platform, we successfully monitored and quantified tumor-induced angiogenesis, and following treatment with antibiotics, observed significant suppression. This deep tissue and large-volume PAM platform provides enhanced 3D insights into the effects of antibiotics on angiogenesis, paving the way for more precise in vitro evaluations of therapeutic interventions and drug screening studies.

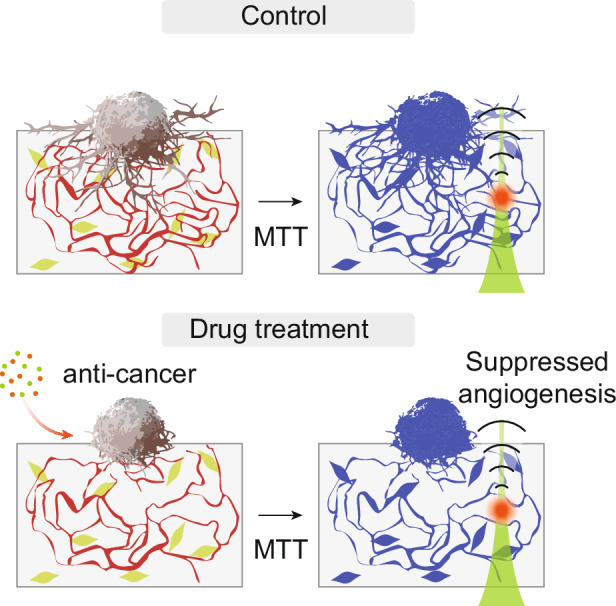

## Introduction

By supplying oxygen and nutrients that support cancer cell survival and proliferation, angiogenesis plays a pivotal role in tumor progression^1–4^. In consequence, inhibiting vascular growth has emerged as a promising cancer treatment strategy, aiming to starve tumors by cutting off their nutrient and oxygen supply^5–9^. To evaluate such anti-angiogenic therapies, in vivo animal models, such as mouse xenografts, have traditionally been employed, due to their physiological relevance. However, these models are associated with low throughput, high cost, ethical concerns, and inter-animal variability, making them less suitable for large-scale or rapid drug screening.

To overcome these drawbacks, in vitro platforms have gained traction, as conventional two-dimensional (2D) cultures are simple and scalable, but fail to reproduce the three-dimensional (3D) cellular architectures and cell–cell/cell–matrix interactions of the native tumor microenvironment^10,11^. To address this issue, in vitro 3D models, including organoids and bioprinted constructs, have emerged as promising platforms^12^. More recently, bioprinted 3D tumor–vessel constructs have emerged^13–16^, to enable reproducible placement of cancer spheroids adjacent to endothelial channels, with precise control over cell ratios and matrix composition^17,18^. These models allow the reconstruction of physiologically relevant tumor–vessel structures in a reproducible and ethical manner, bridging the gap between simplistic 2D assays and complex in vivo studies for drug evaluation. However, visualizing angiogenic sprouts deep within these dense, 3D constructs remains challenging, standard fluorescence and confocal imaging are limited by penetration depth and light scattering. This bottleneck highlights the need for alternative modalities, such as photoacoustic microscopy (PAM), which can deliver volumetric, deep tissue visualization in optically opaque bioprinted tissues.

PAM represents one of the most promising label-free imaging modalities to visualize 3D vascular structures in biological tissues. Unlike optical imaging techniques, such as confocal microscopy, which suffer from light scattering and limited penetration depth^19–23^, PAM offers improved imaging performance in deeper tissues. This is due to the low acoustic attenuation in biological tissues^24–26^, and clear visualization of vascular structures at greater depth. Furthermore, the fast vertical readout of ultrasound signals facilitates efficient 3D imaging of large vascular volumes, providing detailed insight into angiogenic processes. These capabilities establish PAM as a powerful tool to evaluate angiogenesis in bioprinted tumor–vessel models. However, in engineered constructs such as bioprinted tissues or artificial vessel networks, the absence of hemoglobin poses a critical limitation, depriving PAM of its primary endogenous contrast source, and thereby hampering effective visualization. This underscores the need for biocompatible contrast enhancement strategies to unlock the full potential of PAM in synthetic tissue environments.

**Fig. 1:**
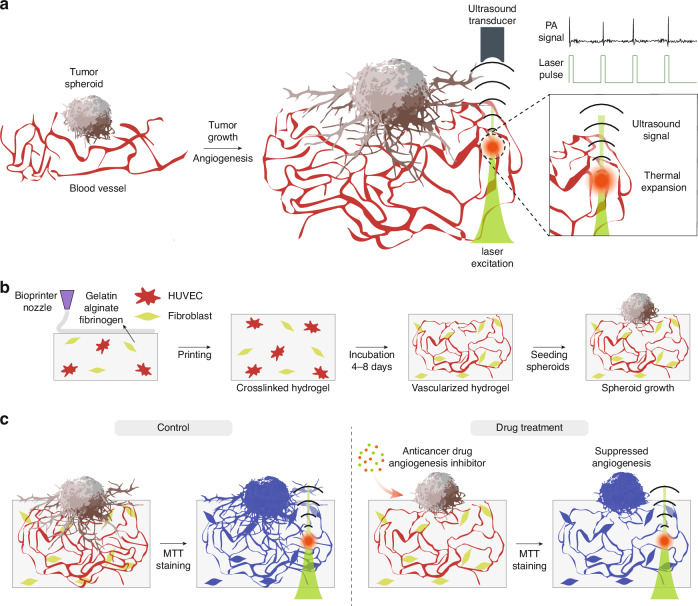
**Schematic of the overall experimental workflow**. **a** Principle of PAM imaging to visualize tumor-induced angiogenesis. **b** Process for generating 3D vascular networks with tumor spheroid seeding in bioprinted hydrogels. **c** Schematic of the PAM-based anti-cancer drug screening platform. Notably, drug treatment significantly suppresses tumor-induced angiogenesis

Herein, we employ a high-resolution photoacoustic microscopy (HR − PAM)-based approach to monitor 3D angiogenesis in bioprinted tumor–vessel model under anti-cancer treatment conditions, enabling rapid and deep tissue imaging with minimized scattering (Fig. 1). HR − PAM reconstructs 3D high-resolution vascular structures by detecting ultrasound waves generated through the thermoelastic expansion of light-absorbing molecules under pulsed laser excitation (Fig. 1a)^27–29^. To address the lack of intrinsic optical contrast in bioprinted vessel structures, we incorporated MTT (3–(4,5–dimethylthiazol–2–yl)–2,5–diphenyltetrazolium bromide) staining into our imaging workflow. Although MTT is traditionally used to assess cell viability, its metabolic conversion into water-insoluble MTT formazan crystals enables strong optical absorption within the 500 − 700 nm range, which is highly suitable for photoacoustic (PA) signal generation^30^. This approach offers broad compatibility for the PA imaging of metabolically active cells. We first characterized the performance of our custom HR − PAM setup. Notably, the 1/e^2^ penetration depth of HR − PAM was ~1.6 times greater than that of confocal microscopy. To model angiogenesis, we bioprinted hydrogels containing human umbilical vein endothelial cells (HUVECs) and human lung fibroblasts (LFs), which spontaneously form 3D vascular networks (Fig. 1b). Tumor spheroids were then seeded on top of the hydrogels, followed by treatment with anti-cancer drugs to evaluate their inhibitory effects on angiogenesis. We employed MTT staining, which labels metabolically active cells by producing dark formazan crystals that provide strong contrast in HR − PAM imaging (Fig. 1c). The HR − PAM images revealed significant suppression of angiogenesis throughout the entire hydrogel depth, with the most pronounced inhibition observed near the tumor spheroids. These results demonstrate that our HR − PAM-based approach enables effective volumetric evaluation of drug effects on tumor-induced angiogenesis in 3D in vitro models.

## Results

### Optical characterization of PAM

#### Measurements of imaging penetration depth

**Fig. 2:**
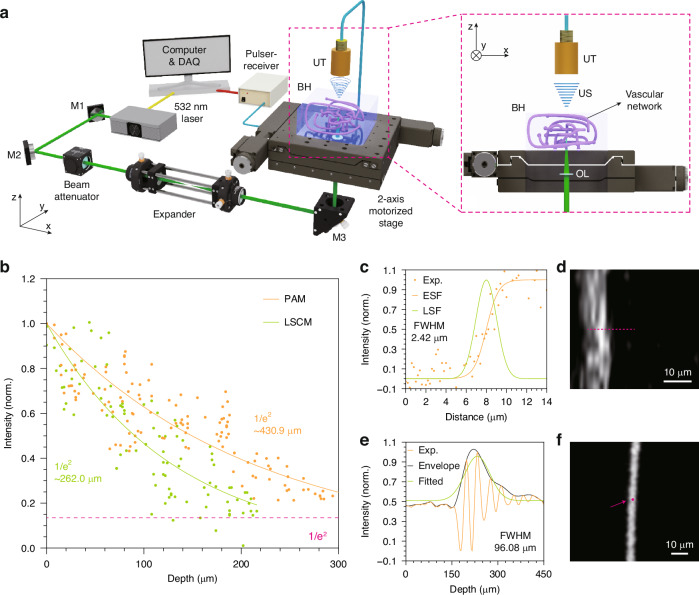
**Characterization of the custom transmission-mode HR** **−** **PAM system**. **a** Schematic of the HR − PAM system. The inset outlined by the dashed magenta line represents the cross-sectional view of the sample stage. M1 − 3, mirrors; UT, ultrasound transducer; BH, bioprinted hydrogel; US, ultrasound; OL, objective lens. **b** Comparison of 1/e^2^ imaging penetration depth between LSCM (green) and PAM (orange) in opaque bioprinted hydrogels embedded with cancer cells. ESF (orange) and corresponding LSF (green) of a razor blade (**c**), along with its PAM image (**d**), used for lateral resolution measurement. The ESF intensity profile was extracted along the magenta dashed line shown in (**d**). Amplitude profile of PA signal (orange) and its envelope (black) from a carbon fiber (**e**), and the corresponding PAM image (**f**), used for axial resolution measurement. The amplitude profile was obtained at the magenta dot indicated by the arrow in (**f**)

We built a custom-made transmission mode HR − PAM system to investigate the effect of anti-cancer drugs on bioprinted tumor–vessel models (Fig. 2a and the Experimental section). The pulsed laser at 532 nm was used for excitation due to its strong absorption by dark formazan crystals, which are specifically formed in metabolically active cells through MTT staining^30^. Although the bioink used for bioprinting is intrinsically transparent, it becomes opaque after loading cells due to the refractive index mismatch between the hydrogel and the embedded cells. This mismatch causes light scattering, which disrupts optical imaging, particularly in deeper regions (Fig. S1). In consequence, it becomes challenging to obtain clear structures using conventional light-based microscopy. To evaluate achievable imaging depth in thick ( > 0.5 − 1 mm), opaque, and inhomogeneous bioprinted hydrogels (Fig. S1)^18^, we first measured the 1/e^2^ penetration depth of our HR − PAM system in the tumor–vessel models. For comparison, we also measured the penetration depth using laser scanning confocal microscopy (LSCM). We prepared bioprinted hydrogels embedded with human glioblastoma cancer cell line U87 MG (U87), using a bioink composed of gelatin, alginate, and fibrinogen to assess the imaging penetration depth. The resulting constructs appeared opaque and had a thickness of ~500 µm. The cancer cells were then stained with MTT and Phalloidin solution to provide contrast for PAM and LSCM, respectively. The penetration depth was estimated by measuring the intensity attenuation of the stained cancer cells with respect to the imaging depth (Fig. S2). Considering staining variance, we manually segmented the stained cells, and obtained median intensity values. The results showed exponentially decaying intensity curves as a function of depth, demonstrating the 1/e^2^ penetration depth of PAM ( ~ 431 µm) was 1.6 times longer than that of LSCM ( ~ 262 µm) (Fig. 2b). These values indicate lower signal attenuation in PAM compared to optical microscopy, when imaging in scattering media. The comparison between the two imaging modalities showed that PAM maintained relatively uniform intensity across the imaging depth compared with confocal microscopy. The scanless axial data acquisition of PAM enables rapid depth-wise imaging; however, its fixed optical focus and limited acoustic resolution result in image blurring. In contrast, confocal microscopy provides clearer images but requires axial scanning, which results in slower depth-wise data acquisition (Fig. S2).

To further characterize the performance of our HR − PAM, we experimentally quantified both lateral and axial resolutions. The system implements an optical-resolution imaging scheme, in which tightly focused light defines the excitation volume. Accordingly, the lateral resolution is governed by the optical diffraction limit, whereas the axial resolution is primarily determined by the bandwidth and center frequency of the ultrasound transducer. To estimate the lateral resolution, we acquired the edge spread function (ESF) using a razor blade, and derived the corresponding line spread function (LSF) by differentiating the ESF. The full width at half maximum (FWHM) of the LSF was measured to be 2.42 µm, which closely matches the theoretical diffraction limit for a wavelength 532 nm and an effective numerical aperture (NA) of 0.12, as calculated in Eq. (1) (Fig. 2c and d)^31,32^:

1 $${R}_{{lateral}}=0.51\frac{\lambda }{{NA}}$$

where, $\lambda$ and ${NA}$ represent the wavelength and numerical aperture, respectively. The axial resolution was assessed by analyzing the axial amplitude profile of a carbon fiber of ~6 µm diameter. A Gaussian function was fitted to the upper envelope of the profile, and the FWHM was measured to determine the resolution, which was found to be ~96.1 µm. The theoretical axial resolution was calculated using Eq. (2)^33^:

2 $${R}_{{axial}}=0.88\frac{v}{B}$$

where, $v$ and $B$ denote the speed of ultrasound in water (1540 m/s) and the bandwidth of the ultrasound transducer, respectively. The measured ultrasound bandwidth, obtained from the pulse–echo response curve (Fig. S3), was approximately 69%, with a center frequency of ~19 MHz. Based on these parameters, the calculated axial resolution was ~103.4 µm, which is in close agreement with the experimentally measured axial resolution ( ~ 96.1 µm) of the HR − PAM (Fig. 2e and f).

### Vascular growth in bioprinted hydrogels

#### 3D PA imaging of angiogenesis in bioprinting hydrogels

**Fig. 3:**
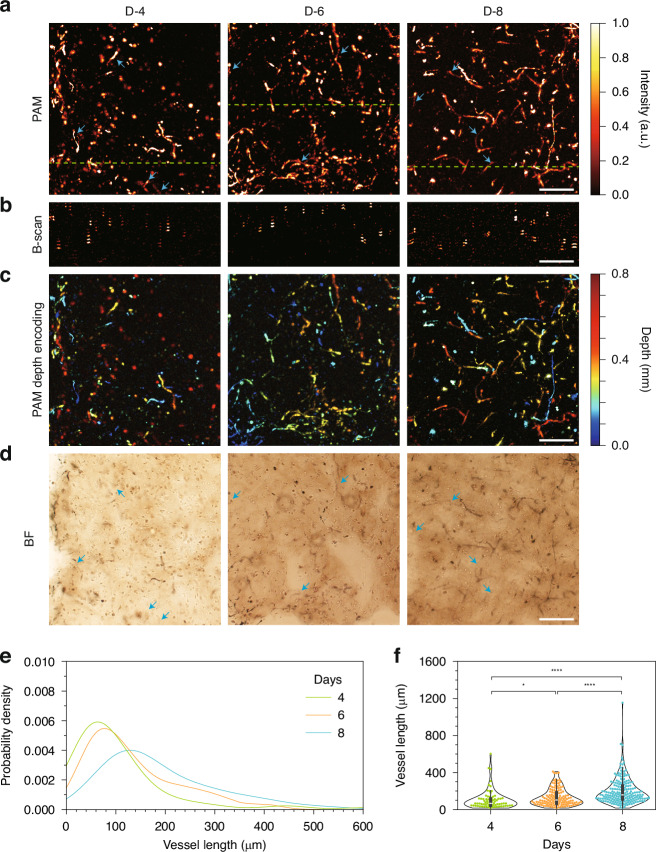
**PAM imaging of time-dependent vascular maturation**. MAP images (**a**), corresponding B-scan images (**b**), depth-encoded projections (**c**), and BF images (**d**) of vascular networks in bioprinted hydrogels cultured for 4, 6, and 8 days. The vascular structures were stained with MTT solution. The B-scan images in (**b**) were acquired along the green dashed lines shown in (**a**). Blue arrows indicate vascular structures that are clearly identified in PA images, but appear ambiguous in BF images. **e** Probability density function of vessel lengths for each culture period. **f** Violin plots showing the distribution of individual vessel lengths. Kruskal–Wallis non-parametric test followed by Dunn’s post hoc test with Bonferroni correction was used for multiple comparisons, as normality was not satisfied in all groups. ****p < 0.0001; ***p < 0.001; **p < 0.01; *p < 0.05; ns, non-significance. Scale bars: 400 µm (**a**–**d**)

To visualize angiogenic progression in a physiologically relevant 3D microenvironment, we employed HR − PAM to monitor vascular development in bioprinted hydrogels over time. Bioprinted hydrogel blocks (10 mm × 10 mm × 1 mm) containing HUVECs and LFs were fabricated using a bioink composed of gelatin, alginate, and fibrinogen. The cell-loaded hydrogels were incubated in EGM − 2 medium under standard cell culture conditions (37 °C, 5% CO_2_ incubation) for up to 2 − 8 days to promote in situ capillary formation. To enhance optical absorption contrast for PA imaging, vascular structures were stained with MTT, which selectively accumulates dark formazan crystals in metabolically active cells. The 3D PA images were acquired using our HR − PAM system (Fig. 3a). Maximum amplitude projection (MAP) images (Fig. 3a) revealed a clear time-dependent increase in vascular complexity, with sparse and fragmented microvessels observed at day 4, followed by progressive elongation and interconnection of vascular networks at day 6 and day 8. The corresponding B-scan images (Fig. 3b), taken along the green dashed lines in MAP views, further confirm the presence of depth-embedded capillaries, many of which are not discernible in conventional bright-field (BF) microscopy, due to the limited depth of field (Figs. 3d and S4). Depth-encoded PA projections (Fig. 3c) clearly visualize the 3D spatial distribution of vasculature, highlighting vessels at varying depths that are otherwise blurred or invisible in BF images (Fig. S4). The blue arrows in Fig. 3d indicate regions where vascular structures were ambiguous or indistinct in BF, but were readily resolved in PA images (corresponding blue arrows in Fig. 3a). Notably, PA imaging provided strong contrast for vascular structures, even at ~0.8 mm depth (Fig. 3b and c) within the thick, inhomogeneous, and opaque cell-loaded hydrogels (Fig. S1). These results highlight the advantage of HR − PAM to image microvascular networks in optically scattering bioprinted constructs, allowing quantitative and depth-resolved analysis of angiogenesis.

#### Quantitative analysis of vascular formation

To quantitatively assess capillary morphogenesis over time, we measured the lengths of individual capillary segments in 3D space using Simple Neurite Tracer (SNT)^34^, an open-source tool to trace 3D networks. Vascular structures segmented from the PA images were traced in three dimensions to compute individual vessel lengths. The resulting vessel length distributions showed a progressive shift toward longer capillaries with increasing culture duration (Fig. 3e). Specifically, the probability density function curves showed that the peak (mode) vessel lengths increased from shorter segments at day 4 to markedly longer segments by day 8, reflecting ongoing vascular maturation.

Statistical comparisons of vessel lengths across time points (Fig. 3f) revealed significant differences in the degree of capillary elongation. The median vessel lengths increased from 67.1 to 103.2 µm at day 4 to 6, and further to 158.5 µm at day 8. Notably, while the maximum vessel lengths remained below 1 mm at earlier time points (602.5 and 410.4 µm at day 4 and 6, respectively), capillaries exceeding 1 mm in length (maximum 1154.7 µm) were observed by day 8, indicating substantial vascular outgrowth. These results highlight the temporal dynamics of angiogenesis in bioprinted hydrogels and validate the ability of PA imaging to quantitatively capture 3D vascular development. The findings are consistent with prior reports of culture time-dependent capillary elongation^18^, and further demonstrate the utility of HR − PAM as a non-invasive modality to monitor vascular maturation in engineered tissue environments.

### Tumor-induced angiogenesis model to evaluate drug responses

#### Tumor spheroid seeding on capillary-formed hydrogels

To establish a model to evaluate anti-cancer drug responses, we seeded U87 glioblastoma spheroids to mimic a tumor-induced angiogenesis environment in bioprinted hydrogels^18^. The spheroid-seeded hydrogels were further cultured for 4 additional days to enable tumor cell invasion into the vascular layer, and to promote tumor-associated angiogenesis within the 3D matrix (Fig. S5). This model recapitulates key features of the tumor–vessel interface, and serves as a platform for preliminary evaluation of drug effects on both cancer progression and angiogenesis. To demonstrate the applicability of this platform to drug response assessment, we tested two clinically relevant anti-cancer agents with distinct mechanisms of action: temozolomide (TMZ), a DNA–alkylating agent that induces apoptosis and inhibits cell proliferation^18,35–40^, and sunitinib (SU), a multi-targeted tyrosine kinase inhibitor that suppresses angiogenesis by blocking vascular endothelial growth factor receptor (VEGFR) and platelet-derived growth factor receptor (PDGFR) signaling^41–45^. Given their complementary effects, a combination treatment of TMZ and SU was expected to exert enhanced anti-tumor efficacy, compared to monotherapy. After drug administration, the hydrogels were incubated for an additional 3 days, followed by MTT staining to visualize metabolically active regions. PA imaging was subsequently performed to assess vascular and tumor responses under each treatment condition.

#### Quantification of 3D vessel structures to investigate drug effects in angiogenesis

**Fig. 4:**
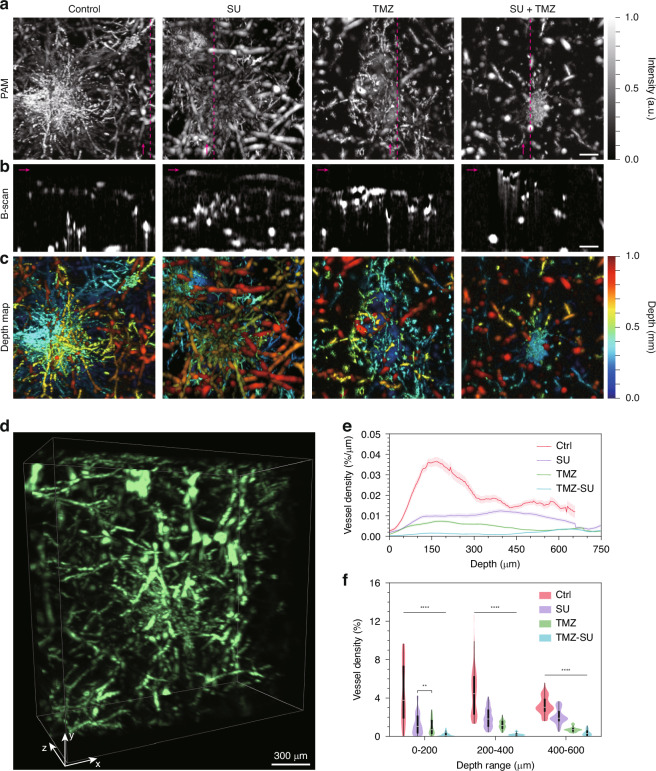
**PAM-based drug screening through 3D quantification of vascular networks**. MAP images (**a**), corresponding B–scan images (**b**), and depth-encoded projections (**c**) of vascular networks in bioprinted hydrogels treated with anti-cancer drugs, including TMZ, SU, and their combination. The B–scan images in (**b**) were acquired along the magenta dashed lines shown in (**a**), with magenta arrows indicating the direction of the scan. **d** Representative 3D reconstruction of vascular networks in SU-treated hydrogels. **e** Comparison of depth-dependent vessel densities with or without drug treatment. **f** Statistical analysis of vessel densities across different depth ranges under various drug treatment conditions. Kruskal–Wallis non-parametric test followed by Dunn’s post hoc test with Bonferroni correction was used for multiple comparisons, as normality was not satisfied in all groups. ****p < 0.0001; ***p < 0.001; **p < 0.01; *p < 0.05; ns, non-significance. Scale bars: 200 μm (**a**–**c**)

MAP images from HR − PAM (Fig. 4a) clearly show the extent and complexity of vascular networks surrounding tumor spheroids. In the control group, dense, radially expanding microvessels were observed, indicating active tumor-induced angiogenesis. In contrast, SU and TMZ monotherapies exhibited partial suppression of capillary growth, with reduced density and branching complexity. The combination of SU and TMZ showed the strongest inhibitory effect, with sparse, fragmented vessels, and minimal angiogenic outgrowth (Fig. S5). These trends are also consistent with fluorescent images (Fig. S6), supporting the findings observed in HR − PAM images. Corresponding B-scan images (Fig. 4b), taken along the magenta lines shown in the MAPs, provided cross-sectional views of the vascular architecture. These images revealed that while MAPs captured complex vessel networks, B-scans enabled depth-resolved visualization, allowing individual vessels within densely packed regions to be resolved. Depth-encoded projections (Fig. 4c) further illustrate the spatial distribution of vasculature throughout the hydrogel volume, highlighting the dramatic reduction in vessel infiltration depth in drug-treated groups. Notably, HR − PAM enabled depth-resolved imaging of vascular structures down to ~1 mm, even within the highly scattering, optically opaque bioprinted tumor–vessel constructs (Fig. S1). This deep tissue imaging capability allowed for comprehensive volumetric characterization of angiogenesis in 3D (Fig. 4d). To compare HR − PAM with conventional imaging, we examined BF images of the same samples (Fig. S7). Although the general vascular outlines were faintly visible in BF mode, the densely packed vessel regions near the spheroids appeared dark and indistinct, particularly in control samples with active angiogenesis. Moreover, the BF images lacked any depth information and failed to distinguish individual vessels within overlapping regions. Merged BF and PAM images (Fig. S7, bottom row) clearly demonstrated the superior resolving power of PAM.

To quantitatively assess vessel formation, 3D vascular networks were reconstructed from HR − PAM data using the MATLAB tool ‘Volume Segmenter’^46,47^. Automated thresholding using Otsu’s method was initially applied to generate vessel masks, followed by manual refinement to correct segmentation errors. The segmented masks were then integrated layer-by-layer to quantify vascular volume and density as a function of depth.

As shown in Fig. 4e, the control group exhibited the highest vessel density, peaking at shallow depths (0 − 150 µm) near the tumor spheroid location. SU and TMZ monotherapies resulted in noticeable suppression of vascular growth, particularly in superficial regions. The combination therapy of TMZ and SU exhibited the strongest suppression across all depths. Statistical comparison confirmed these trends, showing significant reductions in vessel density at all measured depth ranges (0–200, 200–400, and 400–600 μm), with the combined drug group showing the lowest values (Fig. 4f). Vessel density was also reflected in vessel complexity, as frequent branching results in a dense and complex vascular network. The depth-dependent fractal dimension, a measure of vessel complexity^48–50^, shows a trend consistent with vessel density (Fig. S8). These results demonstrate the ability of HR − PAM to visualize and quantify complex angiogenic responses in 3D. Moreover, the comparison with BF imaging highlights the unique advantages of PAM in capturing deep, densely packed vascular structures at high resolution, making it an effective tool for evaluating anti-angiogenic drug effects in engineered tumor–vascular models.

## Discussion

Angiogenesis is a hallmark of cancer progression, because it provides tumors with the oxygen and nutrients needed for continued growth^1–4,51–53^. Consequently, inhibiting angiogenesis has emerged as a promising therapeutic strategy by restricting nutrient supply to tumor tissues, and thereby limiting proliferation^5–9,54–60^. Quantitative analysis of angiogenesis plays an important role in monitoring tumor development and evaluating therapeutic responses, particularly for drugs targeting vascular formation. However, existing in vitro approaches often fail to mimic the complex 3D architecture of native vasculature^10,11^, while in vivo models, despite their physiological relevance, are constrained by high costs, ethical considerations, limited throughput, and limited imaging depth^13,14,61–63^.

To address these limitations, we developed a bioprinted tumor-induced angiogenesis model and demonstrated the utility of HR − PAM for 3D visualization and quantification of angiogenic responses under anti-cancer drug treatment. This platform combines the architectural complexity and scalability of bioprinting with the deep tissue imaging and volumetric capabilities of PAM, offering a balanced alternative between conventional in vitro and in vivo approaches. PAM detects ultrasound signals generated by the thermoelastic expansion of absorptive structures upon pulsed laser excitation. Compared to optical modalities, such as confocal microscopy, our custom-built transmission-mode HR − PAM achieved a ~ 1.6-fold greater 1/e² penetration depth, which is attributed to the low acoustic attenuation in biological samples. This enabled depth-resolved imaging across the full thickness ( ~ 1 mm) of the bioprinted hydrogels, facilitating the accurate 3D reconstruction of vascular structures with minimal signal loss. It is important to note that this millimeter scale full-thickness imaging was achieved without axial scanning, owing to the ultrasound-based readout. The rapid acquisition of ultrasound signals allowed high-speed volumetric imaging across large fields of view, making it suitable for scalable drug response analysis. While the current system provides scanless 3D imaging approximately 1 mm in depth, this range can be further extended by incorporating axial scanning or employing high aspect ratio optical beams such as Bessel^64^ or needle beams^65,66^ in transparent media. Specifically, combining additional axial scanning with needle beam illumination can increase the overall imaging depth beyond the millimeter scale. In highly scattering tissues, acoustic-resolution PAM can be utilized to achieve centimeter scale imaging at the cost of spatial resolution.

While BF imaging showed partial visualization of vascular morphology, it lacked depth information and failed to resolve individual capillaries in regions of high vessel density. In contrast, HR − PAM offered fine structural details and clear vessel boundaries throughout the entire volume. Merged PAM − BF images further highlighted this difference, with PAM clearly delineating complex vascular networks that appeared indistinct or overlapped in BF images. Time-dependent PA imaging also enabled longitudinal monitoring of angiogenic progression, revealing significant increases in both mode and maximum vessel lengths from day 4 to day 8 of culture, indicative of capillary maturation. These results align with prior reports on in vitro angiogenesis^18^, confirming the validity of our quantification pipeline.

We also demonstrated the applicability of our platform for evaluating anti-cancer therapeutics by seeding tumor spheroids onto capillary-formed bioprinted hydrogels and applying TMZ, SU, or their combination. These drugs target tumor proliferation via distinct mechanisms, TMZ through DNA alkylation and apoptosis, and SU through the inhibition of tyrosine kinase-mediated angiogenesis. Quantitative analysis of depth-resolved vessel density revealed substantial inhibition of angiogenesis in all drug-treated groups, with the combination therapy yielding the most pronounced effect. Notably, the suppression was most significant near the tumor spheroid seeding region (0−200 µm), but the inhibitory trend persisted across the entire 3D volume, underscoring the importance of volumetric imaging in depth-dependent drug responses.

Together with these results, it is important to note that while PAM enables deep tissue imaging of vascular structures, it inherently suffers from limited axial resolution, which restricts accurate 3D reconstruction of fine structures. To mitigate this limitation, various hardware- and software-oriented strategies, such as the use of high-frequency transducers^67^, deconvolution and deep learning-based restoration^68,69^, and synthetic aperture focusing techniques (SAFT)^70^, have been widely adopted to effectively enhance the resolving power.

PAM inherently supports label-free imaging by detecting optical absorption from endogenous chromophores, such as hemoglobin, melanin, cytochromes, and nucleic-acid, each exhibiting distinct wavelength-dependent absorption. By tuning the excitation wavelength, these intrinsic absorbers can be selectively visualized, enabling label-free PA imaging of various biological tissues. However, our bioprinted vascular model lacked intrinsic blood flow and thus did not contain hemoglobin, the major absorber for label-free imaging. To overcome this, we employed MTT staining to enhance image contrast by targeting metabolically active cells. While effective, MTT provides non-specific labeling and cannot capture functional dynamics such as perfusion or oxygenation. Nonetheless, PAM remains highly effective for label-free imaging in clinical or preclinical studies where intrinsic absorbers are present, and staining is not feasible, providing structural and molecular information under appropriate excitation conditions. While not all biological targets can be visualized without labeling, this intrinsic contrast mechanism makes PAM a powerful tool for noninvasive imaging in deep, scattering tissues. Future advancements for our bioprinting model could include the incorporation of genetically encoded or molecular contrast agents, blood-mimicking perfusion systems, or multi-wavelength functional PAM to enable cell-type-specific and functional vascular imaging.

In conclusion, our integrated bioprinting-PAM platform provides a physiologically relevant, scalable, and quantitative system to evaluate tumor angiogenesis and anti-cancer drug efficacy in 3D. It addresses key limitations of current models by combining tissue-mimicking architecture with deep tissue and high-resolution imaging. While limitations remain, particularly in contrast specificity, the ongoing development of functional imaging techniques and advanced vascular modeling holds promise for future applications. In particular, by incorporating patient-derived cells (PDCs) into the bioprinted tumor–vessel constructs, this platform can be adapted for personalized medicine, enabling patient-specific drug testing and preclinical screening in a biomimetic 3D microenvironment.

## Materials and methods

### Cell culture

HUVECs (ATCC PCS-100-010; RRID: CVCL_2959), the U87 MG glioblastoma cell line (U87) (ATCC HTB-14; RRID: CVCL_0022), and LFs (ATCC PCS-201-013; RRID not available) were acquired from the American Type Culture Collection (ATCC, Bethesda, MD, USA). LFs were included for their supportive role in angiogenesis as our previous study^18^. All cell lines were confirmed to be contamination-free by ATCC. HUVECs at passage five were maintained in endothelial growth medium–2 (EGM − 2; Lonza, Basel, Switzerland) under standard incubation conditions (37 °C, 5% CO₂), and subsequently used in the fabrication of vascularized tissue constructs. LFs at passages five and six were cultured in fibroblast growth medium (FGM − 2; Lonza) under identical conditions. U87 cells were propagated in minimum essential medium (MEM; Life Technologies, Carlsbad, CA, USA), supplemented with 10% fetal bovine serum (HyClone Laboratories, Logan, UT, USA) and 1% penicillin (Life Technologies), and incubated at 37 °C with 5% CO₂.

### Preparation of bioink

Gelatin and alginate were separately dissolved in a 0.9% (w/v) NaCl₂ solution at final concentrations of 20% (w/v) and 4% (w/v), respectively. The two solutions were combined in a 2:1 volume ratio and incubated at 60 °C for 1 h, followed by an additional 2 h at room temperature (RT). Fibrinogen (Sigma–Aldrich, St. Louis, MO, USA) was dissolved in phosphate-buffered saline (PBS, pH 7.4) to prepare a 4% (w/v) solution and incubated at 37 °C for 1 h. HUVECs and LFs were suspended in the fibrinogen solution at a density of 4 × 10⁶ cells/mL. The gelatin/alginate solution was then mixed with the cell-laden fibrinogen solution in a 3:1 ratio, yielding final concentrations of 10% gelatin, 1% alginate, and 1% fibrinogen, with HUVECs and LFs each at a final cell density of 1 × 10⁶ cells/mL.

### Bioprinting of blood vessel layer

The prepared bioink containing HUVECs and LFs was loaded into a 10 mL syringe (HSW, Tuttlingen, Germany) equipped with a 250 μm tip (Nordson EFD, East Providence, RI, USA). The syringe was pre-cooled at 4 °C for 15 min before being mounted onto a bioprinter (INVIVO, ROKIT Healthcare, Seoul, Korea). The dispensing and printing bed temperatures were set to 23 °C and 19 °C, respectively. To promote adhesion between the hydrogel and the Petri dish surface, the dish was treated sequentially with 1% (v/v) polyethyleneimine (PEI) for 30 min, and 0.1% (v/v) glutaraldehyde for an additional 30 min. A cuboidal construct (10 mm × 10 mm × 0.6 mm) was printed in a layer-by-layer manner. Following bioprinting, the construct was incubated with 1 mL of 3% (w/v) CaCl₂ in deionized distilled water (DDW) at RT for 3 min to crosslink the alginate. The construct was then rinsed three times with PBS (pH 7.4). Fibrinogen was crosslinked by adding thrombin (2 U/mL, final concentration) in PBS, and incubating at RT for 15 min. After PBS washing 3 times, the construct was cultured in EGM − 2 at 37 °C in a CO₂ incubator for 7 days to facilitate blood vessel formation.

### Formation of U87 spheroid and seeding onto bioprinted blood vessel layer

U87 cells were freshly harvested and suspended at 3 × 10⁶ cells/mL in 1 mL of medium, before being seeded into concave microwells (853 wells, diameter: 400 μm) (StemFIT3D, Microfit, Seongnam, Korea). Cells were cultured at 37 °C in a CO₂ incubator with daily media exchanges for 3 days, allowing spheroid formation. Four to six spheroids were gently collected from the microwells and seeded onto the constructs, which had first been cultured in EGM-2 medium for 7 days and had their medium removed. To allow spheroids to attach to the bioprinted blood vessel layer, the construct was incubated at 37 °C with 5% CO₂ for 2 h. After spheroid seeding, the hydrogels were further incubated for 4 days to enable cancer cell invasion into the vascular layer and to induce tumor-associated angiogenesis.

### Drug treatment

TMZ and SU were obtained from Sigma–Aldrich. Stock solutions of TMZ and SU were prepared in dimethyl sulfoxide (DMSO). Once spheroids had adhered to the construct, drug treatment was performed using either TMZ (500 μM), SU (50 μM), or a combination of both (TMZ 500 μM + SU 50 μM). The constructions were incubated for 3 days before imaging.

### MTT staining

MTT (3–(4,5–Dimethylthiazol–2–yl)–2,5–Diphenyltetrazolium Bromide) (M6494, ThermoFisher, Waltham, MA, USA) stock solution (5 mg/mL) was prepared by dissolving MTT powder in PBS with sonication to ensure complete dissolution. The stock solution was then diluted in culture media at a 10:1 volume ratio before use. For live cell staining, a bioprinted hydrogel block with or without tumor spheroid was incubated in the diluted MTT solution at 37 °C for 4 h.

### PA imaging

The illumination source was a 532 nm nanosecond pulsed laser (DX − 532 − 2, Photonics Industries International, Ronkonkoma, NY, USA) with pulse repetition rate of 20 kHz and pulse width of 6 ns. The laser power was adjusted by a polarizing beam splitter combined with half-wave plate (VA5 − 532/M, Thorlabs, Newton, NJ, USA). The laser beam was collimated and expanded using a 4 f system to ensure the effective NA of the objective lens (10×, Olympus, Tokyo, Japan) to be 0.12. The laser power was carefully adjusted to ensure the pulse energy was ~15 mJ cm^−2^, which is lower than the American National Standards Institute safety limit for biological safety standards.^65,71^ The manual translation stage (MS1S/M, Thorlabs) with 6.5 mm was used to move the objective lens axially, focusing the laser beam on the sample immersed in a PBS solution.

The PA signal was obtained using a custom-made focused ultrasound transducer with focal length of 11.6 mm (15.7 µs), −6 dB center frequency of 19 MHz, and bandwidth of 69%. The transducer was mounted on a 3-axis translation stage (PT3/M, Thorlabs) for co-focal alignment with the focused laser beam. The collected PA signal was amplified and filtered by pulser/receiver (DPR500, JSR Corporation, Tokyo, Japan) with gain of 27−30 dB and hardware bandpass filter of 12.5 − 30 MHz, respectively. The processed analog signal was converted into a digital signal by 12-bit digitizer (ATS9352, Alazar Technologies, Pointe–Claire, Canada) at a sampling rate of 500 MS/s.

A 2-axis motorized stage (8MTF − 75LS05, Standa, Vilnius, Lithuania) was employed to obtain images with scanning in the horizontal and vertical directions on samples. All trigger signals to adjust laser irradiation, data acquisition, and motor movement were controlled by a multi-function I/O board (PCIE − 6361, National Instruments, Austin, TX, USA). The entire system was operated through custom-developed software (LabVIEW, National Instruments). The speed of sound was set to 1540 m/s to convert the ultrasound echo signal time delay to the axial position, resulting in axial pixel size of 3 µm. The lateral resolution, measured from the edge spread function (ESF) of a razor blade, was 2.42 µm, whereas the axial resolution, obtained from the axial intensity profile of a carbon fiber with a nominal diameter of 10 µm, was 96.1 µm.

### Penetration depth measurements

U87 cells were loaded in bioink at a density of 2 × 10⁶ cells/mL, and bioprinted to compare the penetration depth of PAM and LSCM. For PAM imaging, US87 cell-embedded hydrogel blocks (10 mm × 10 mm × 1 mm) were incubated in a final staining solution, prepared by diluting MTT stock solution (5 mg/mL) in culture media at a 10:1 volume ratio, and maintained at 37 °C for 4 h. For LSCM imaging, additional hydrogel blocks were incubated at RT for 30−60 min in a final staining solution prepared by diluting Phalloidin (R415, ThermoFisher) stock solution (66 µM in DMSO) in PBS at a 1:400 v/v ratio. The MTT and Phalloidin stained cells were imaged using PAM and LSCM, respectively. To qualify the depth-dependent intensity attenuation, the largest cross-section of cells was manually segmented, and the median value was obtained at each depth within the hydrogel block.

### Data processing

All image acquisition was performed using custom-made software with a voxel size set to 2 µm × 2 µm × 3 µm. The acquired images were processed using MATLAB 2024a and Python for 3D reconstruction and quality enhancement. For 3D PAM image reconstruction, the upper envelope of the photoacoustic A–line signals was extracted and assigned to their corresponding pixel positions. A 3D median filter with a window size of 3 × 3 × 3 voxels was applied to the reconstructed data to reduce noise.

## Supplementary information

Supporting Information

## Data Availability

Data is available from the corresponding authors upon reasonable request.

## Code availability

All relevant codes are available from the corresponding author upon reasonable request.
