# Supplementary material for: Photoacoustic microscopy reveals deep angiogenic responses in 3D bioprinted tumor–vessel models
Source: Microsyst Nanoeng. 2026 Apr 13;12:129. doi: 10.1038/s41378-026-01243-y (PMC13076793; doi:10.1038/s41378-026-01243-y)
Supplement: Supplementary file 1 — Supporting Information [file 41378_2026_1243_MOESM1_ESM.docx]

Supporting Information

**Photoacoustic Microscopy Reveals Deep Angiogenic Responses in 3D Bioprinted Tumor–vessel Models**

Yongjae Jo^1, #^, Seokgyu Han^3, 4,^ ^#^, Hyunjun Kye^1^, Jinhee Yoo^1^, Junhyung Kim^2^, Inki Kim^1^, Sungsu Park^1, 2, 3,^ *, and Byullee Park^1, 2,^ *

^#^These authors contributed equally to this work

*Corresponding authors:

Byullee Park: [byullee@skku.edu](mailto:byullee@skku.edu)

Sungsu Park: [nanopark@skku.edu](mailto:nanopark@skku.edu)

^1^ Department of Biophysics, Institute of Quantum Biophysics, Sungkyunkwan University, Suwon 16419, Republic of Korea

^2^ Departments of MetaBioHealth, Biopharmaceutical Convergence, Sungkyunkwan University, Suwon 16419, Republic of Korea

^3^ School of Mechanical Engineering, Sungkyunkwan University, Suwon 16419, Republic of Korea

^4^ Division of Engineering in Medicine, Department of Medicine, Brigham and Women's Hospital, Harvard Medical School, Cambridge, MA, 02139 USA

Keywords: photoacoustic imaging, cancer spheroids, bioprinting, vascularization, drug screening


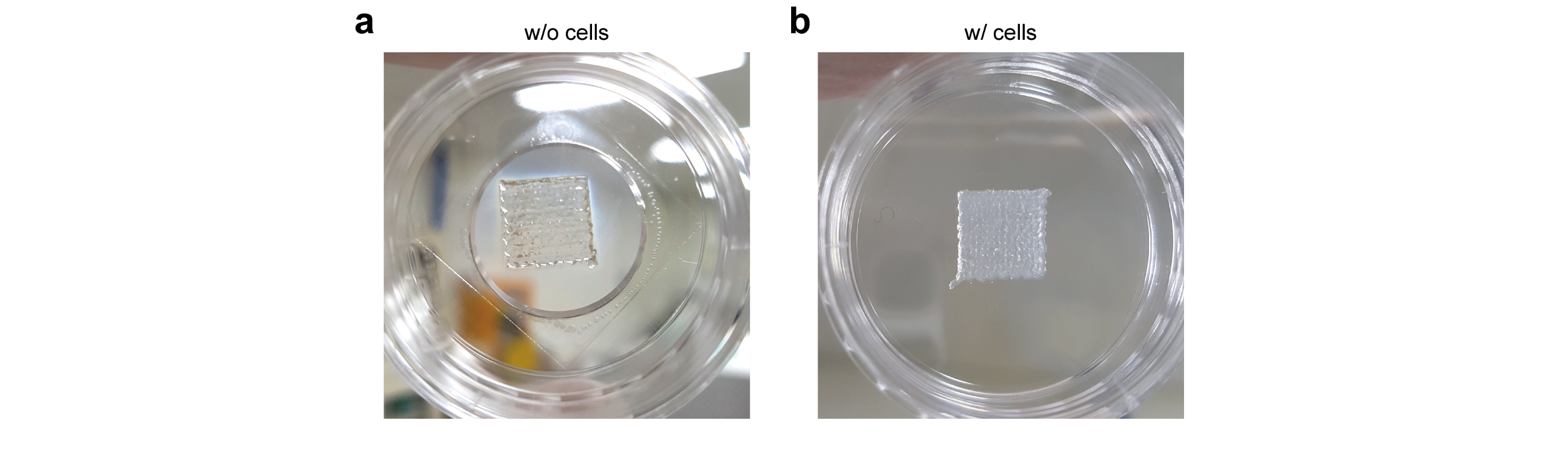


**Figure S1.** **Comparison of optical properties of bioprinted hydrogels. (a, b)** Photographs of bioprinted hydrogels without (a) and with (b) embedded cells. Notably, the cell-embedded hydrogel appeared opaque, due to the refractive index mismatch between the hydrogel and the cells, whereas the hydrogel without embedded cells remained transparent.


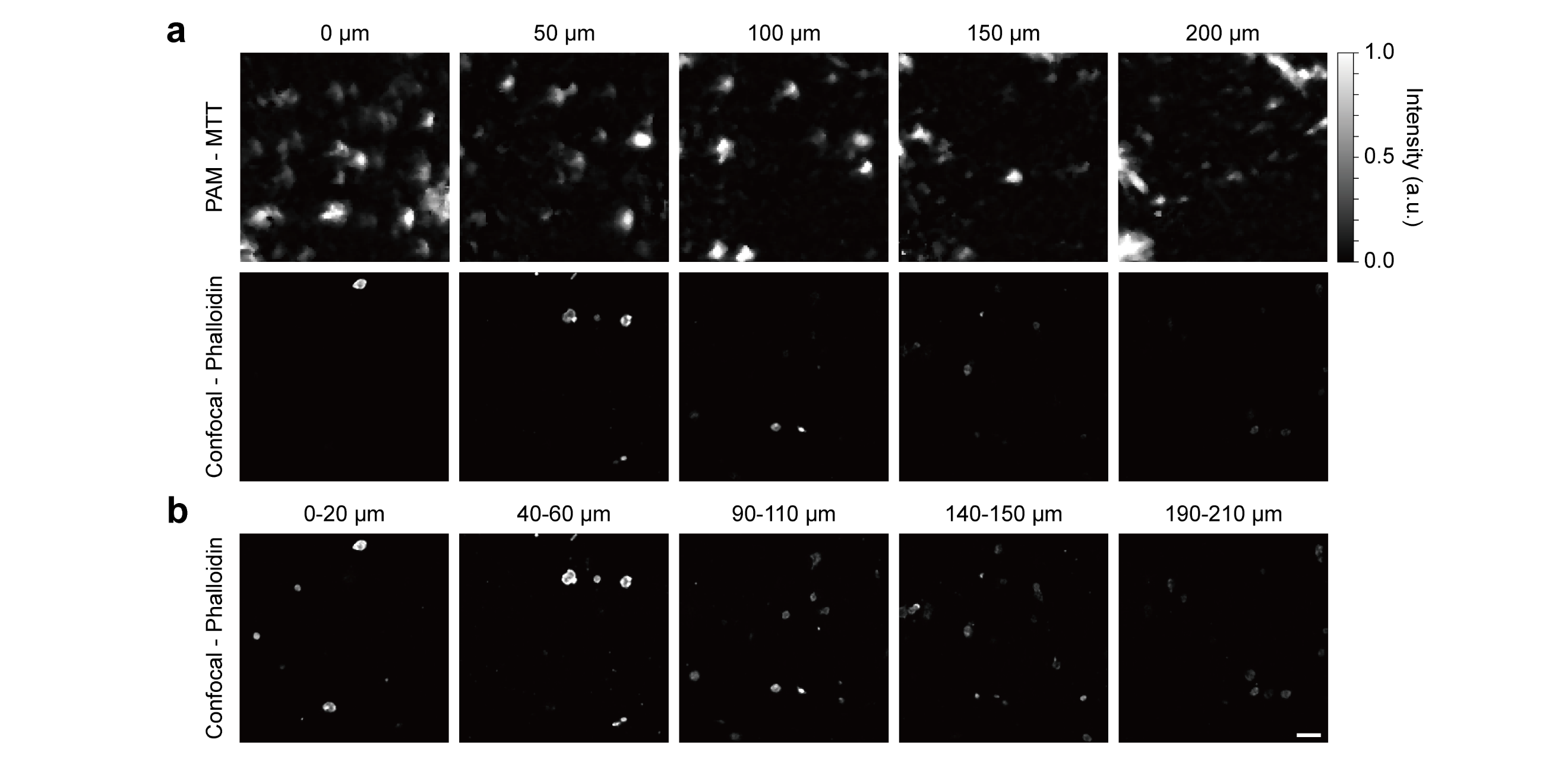


**Figure S2.** **Comparison between PAM and confocal imaging. (a)** Representative PAM (top) and confocal microscopy (bottom) images of U87 cells embedded within hydrogel constructs at various depths. Cells were stained with MTT for PAM and with Phalloidin for confocal imaging. **(b)** Maximum intensity projection confocal images corresponding to the indicated depth ranges from the same sample in (a). Scale bar: 50 µm (a, b).


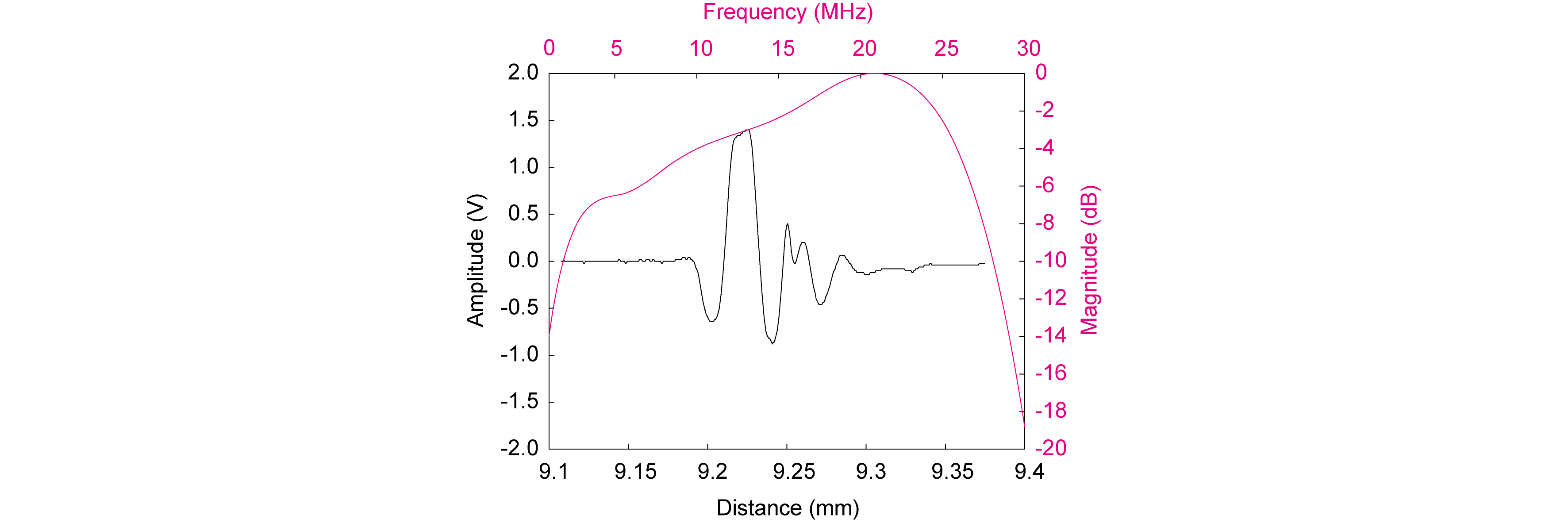


**Figure S3.** **Pulse–echo response of the ultrasound transducer in the PAM system.** Time–domain (black) and frequency–domain (magenta) representations of the pulse–echo response. The transducer exhibited a center frequency of ~19 MHz, and a bandwidth of ~69%.


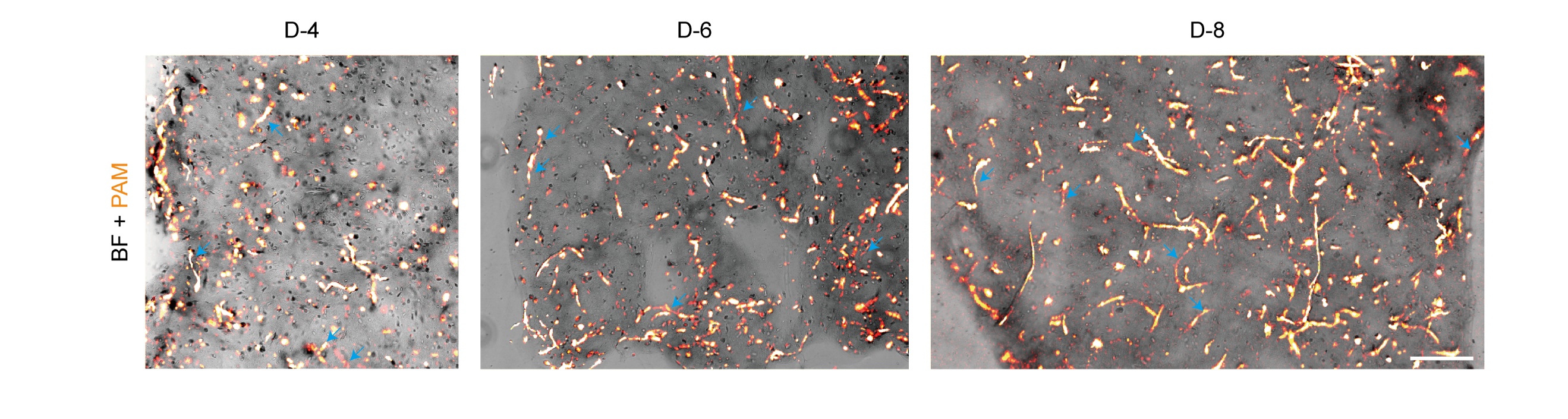


**Figure S4.** **Merged BF and PAM images of vascular networks.** Correlative images of BF (gray lookup table, LUT) and PAM (hot LUT) for hydrogels cultured for 4, 6, and 8 days, comparing the visibility of vascular structures between imaging modalities. Blue arrows indicate vascular structures that are clearly identified in PA images, but appear ambiguous in BF images. Scale bar: 400 µm.


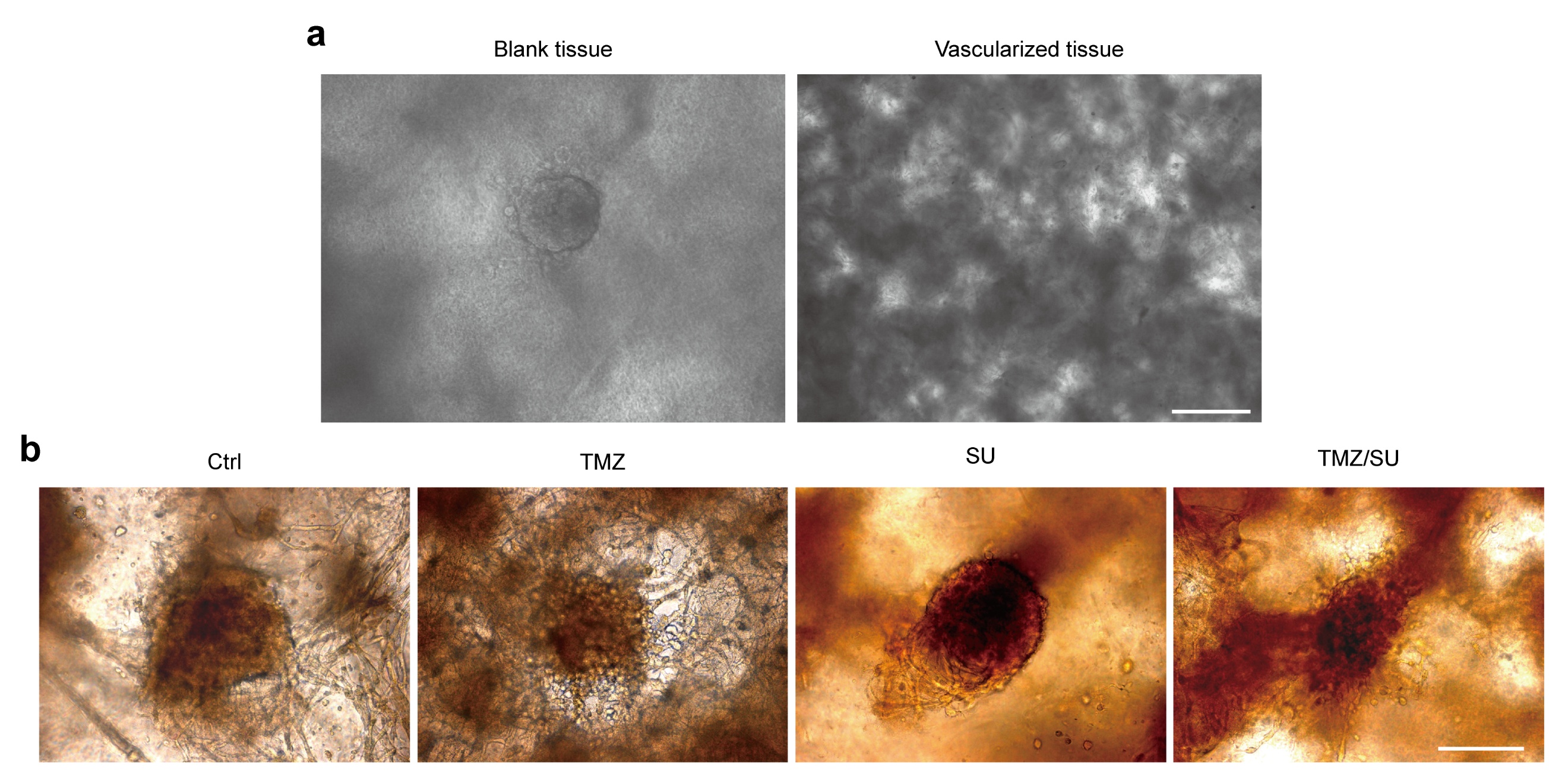


**Figure S5.** **U87 spheroid seeded on vascularized tissue.** **(a)** Bright-field images showing U87 spheroid seeded onto the blank (only gelatin, alginate, and fibrinogen) and vascularized (HUVEC/LF in gelatin, alginate, and fibrinogen) tissues. **(b)** Representative images of U87 spheroids seeded onto vascularized tissues treated without, or with, TMZ, SU, and TMZ/SU combination on day 3 after seeding. Scale bars: 200 µm.


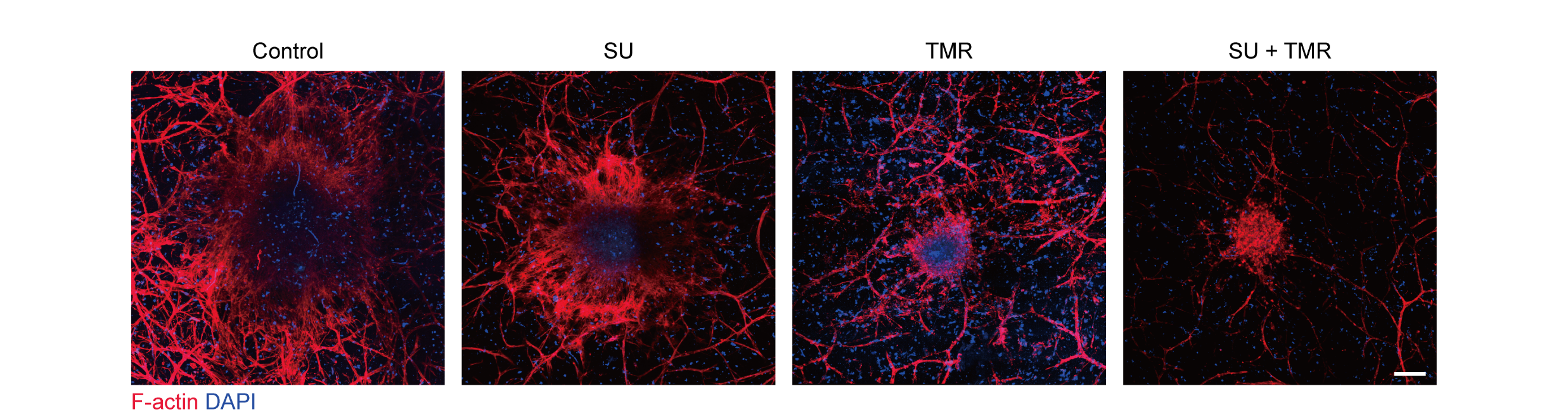


**Figure S6.** **Fluorescence images of vascular networks in tumor-seeded hydrogels under various drug treatments.** Vascular structures were stained with Phalloidin (F–actin) and DAPI (nuclei). Scale bar: 200 µm.


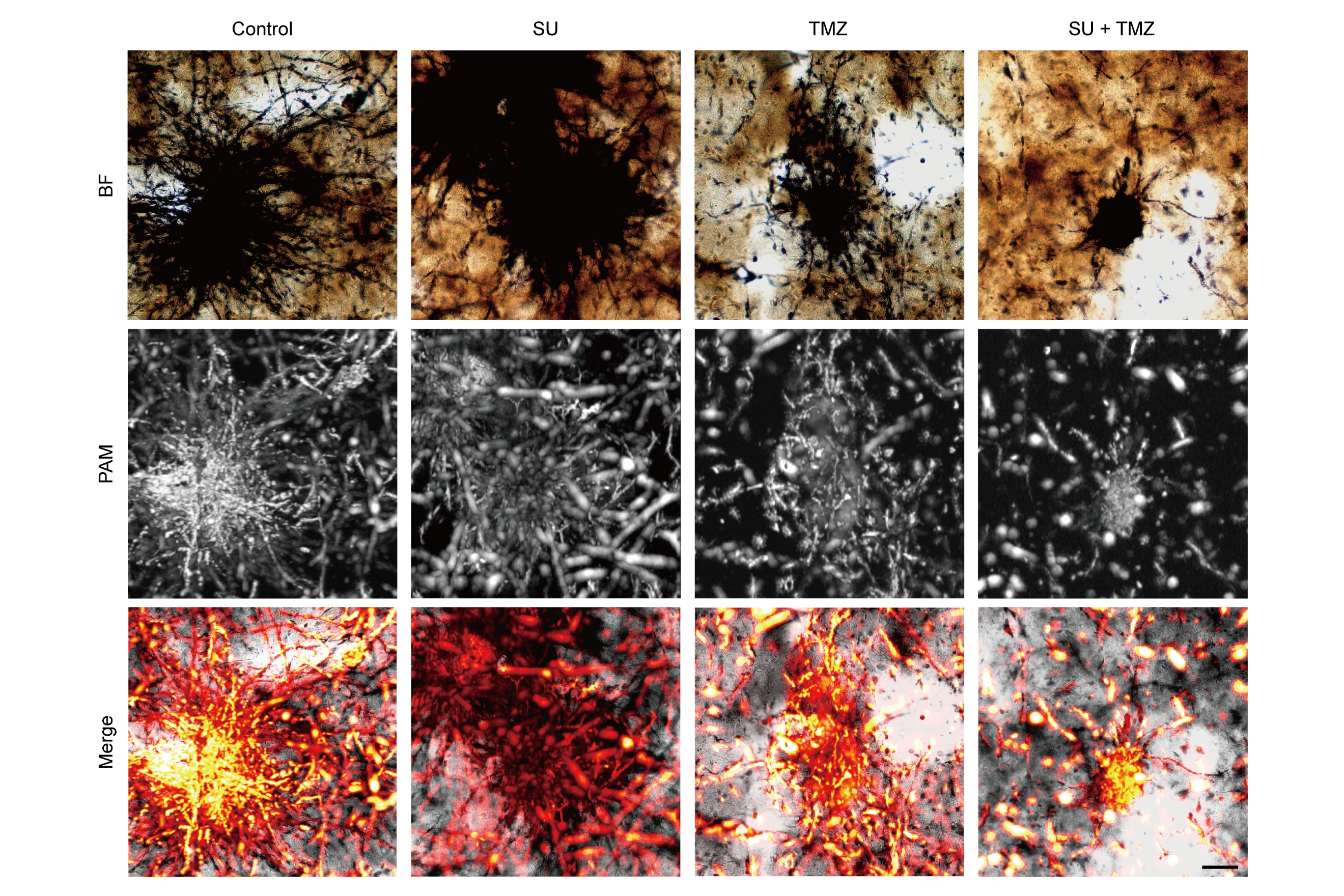


**Figure S7.** **Merged BF and PAM images of vascular networks in tumor-seeded hydrogels under various drug treatments.** BF, MAP of PAM images, and their merged views for the control group and drug-treated groups, including SU, TMZ, and their combination. Scale bar: 200 µm.


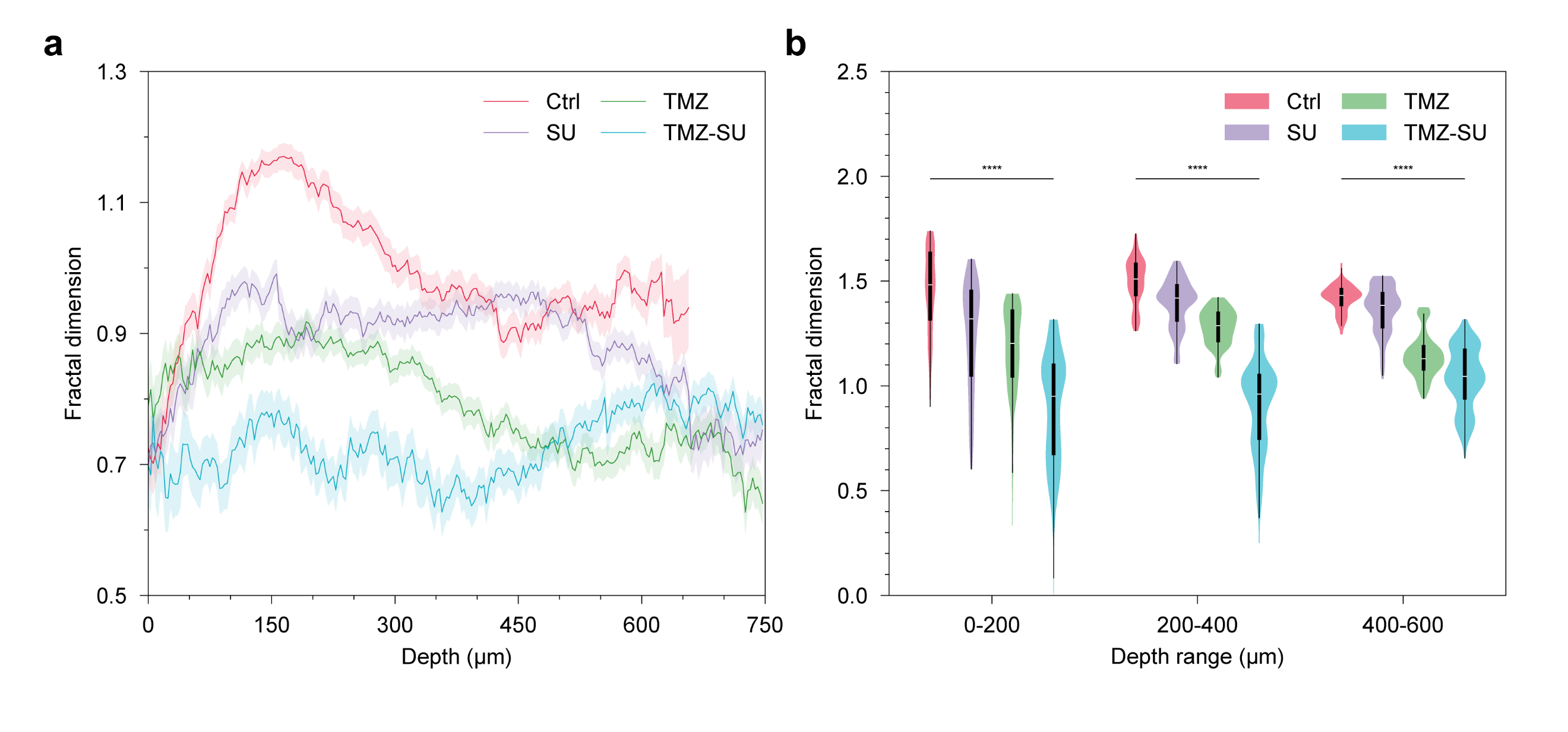


**Figure S8. 3D quantification of vascular network complexity**. **(a, b)** Depth profile (a) and statistical analysis (b) of fractal dimension-based vessel complexity under various drug treatment conditions. As normality was not satisfied in all groups, the Kruskal–Wallis non-parametric test was used, followed by Dunn’s post hoc test with Bonferroni correction for multiple comparisons. *****p* < 0.0001; ****p* < 0.001; ***p* < 0.01; **p* < 0.05; ns, non-significance.
